# Optimization of Flexural Performance of PETG Samples Produced by Fused Filament Fabrication with Response Surface Method

**DOI:** 10.3390/polym16142020

**Published:** 2024-07-15

**Authors:** Oğuz Tunçel, Çağlar Kahya, Kenan Tüfekci

**Affiliations:** 1 Department of Mechanical Engineering, Faculty of Engineering, Siirt University, Siirt 56100, Türkiye; 2 Department of Mechanical Engineering, Faculty of Engineering, Bursa Uludağ University, Bursa 16059, Türkiye; ckahya@uludag.edu.tr (Ç.K.); kenantufekci@uludag.edu.tr (K.T.)

**Keywords:** FFF, petg, response surface methodology, box–behnken design, flexural performance

## Abstract

Additive manufacturing (AM), particularly fused filament fabrication (FFF), has gained significant attention for its design flexibility and cost-effectiveness. This study focuses on optimizing FFF parameters that employ response surface methodology (RSM) to enhance the flexural performance of polyethylene terephthalate glycol (PETG) parts. Three essential parameters—layer height, print speed, and nozzle temperature—were varied, and their effects on flexural strength, flexural modulus, flexural toughness for ultimate strength, flexural toughness at 5% strain, and strain at ultimate strength were evaluated. Based on a Box–Behnken design, the experiments revealed significant effects of these parameters on the mechanical responses. The analysis of variance (ANOVA) indicates that layer height predominantly affects flexural modulus and toughness, while nozzle temperature significantly impacts flexural strength. The RSM models exhibited high accuracy, with R^2^ values exceeding 99%. Optimal parameter combinations yield remarkable improvements: flexural strength reached 39.55 MPa, flexural modulus peaked at 1344.60 MPa, flexural toughness for ultimate strength reached 218.22 J/mm^3^, flexural toughness at 5% strain reached 381.47 J/mm^3^, and strain at ultimate strength reached 3.50%. Validation experiments confirm the effectiveness of the optimization, with errors below 3.17%.

## 1. Introduction

AM is becoming progressively influential in shaping the direction of industry development. The advantages of AM are design flexibility, customization, waste minimization, and the capability to produce complex structures. There has been a significant focus on advancing AM technologies in recent years. There are many different AM technologies available today. One of the most popular and widely used is FFF [1,2,3,4]. FFF is a cost-effective AM method that uses polymer materials. It builds products layer by layer and is widespread for creating components from thermoplastics and fiber-reinforced composites. Current studies examine the quality, build time, surface finish, part quality, mechanical properties, and production costs of parts produced using FFF. Optimal parameter selection affects product quality, and the properties of FFF-produced parts correlate with changing print parameters [5,6].

A wide variety of polymer filaments such as ABS, PLA, Nylon, PC, TPU, and PET can be printed with good dimensional flexibility using FFF. Among these, PET is a widely used thermoplastic. PETG, derived from PET and glycol, boasts attractive mechanical and chemical properties, including robust chemical resistance, considerable impact strength, and material flexibility, owing to its chemical composition. PETG is ideal for AM as it has a lower viscosity than PET. Although PETG demonstrates a higher glass transition temperature (Tg), the mechanical characteristics exhibit similarities, approximately 67 °C [7,8]. Moreover, PETG is suitable for food applications and can be recycled, leading to waste reduction and environmental advantages [9]. However, the 3D printing of polyolefins (e.g., polyethylene and polypropylene) faces challenges due to their high tendency to crystallize. This leads to high-volume shrinkage and interlayer adhesion problems during printing [10]. In particular, PETG, which has a semi-crystalline structure, can experience significant deformation and interlayer adhesion problems during printing [11]. Therefore, the optimization of printing parameters and the improvement of printer designs are required for the development of AM technology.

The parameters set for FFF printing change the part’s mechanical properties and affect the quality. The set of parameters encompasses parameters concerning slicing, build orientation, and temperature. Slicing involves layer height, infill density, infill pattern, nozzle diameter, and print speed. Build orientation determines the placement of the part, while temperature conditions affect adhesion and mechanical properties [12]. Therefore, studies on the influence and optimization of parameters to achieve high-mechanical properties are fundamental. This study has taken flexural performance as an output set to demonstrate the applicability of FFF fabricated parts as end-use parts.

It is possible to access lots of data from existing literature concerning flexural performance, printing parameters, and optimization methodologies applied to specimens fabricated via FFF. In particular, we focused on flexural performance studies using PETG and their results. Gao et al. [13] found that their study comparing the Taguchi and RSM for optimizing FFF parameters revealed differing conclusions on significant factors influencing PLA sample performance. Both methods showed better predictions than the original groups, with RSM providing higher optimum combinations for tensile and compressive strength by 2.11% and 8.15%, respectively. Kumar et al. [14] obtained noteworthy results regarding carbon fiber-reinforced PETG in their investigation. By optimizing machine parameters like print speed, infill density, and layer height, the research attained peak tensile strength (31.57 MPa), flexural strength (35.05 MPa), and hardness (67 BHN). The mentioned machine parameters, such as a print speed of 60 mm/sec, an infill density of 80%, and a layer height of 0.2 mm, provide valuable insights into how to efficiently 3D print PETG material for diverse applications. Valvez et al. [15] conducted a study optimizing printing parameters for enhanced mechanical properties in PETG and fiber-reinforced composites. The results showed flexural stress values of 66.9 MPa for PETG, 79.2 MPa for PETG+CF, and 47.7 MPa for PETG+KF. Temperature significantly influenced filament fluidity and inter-layer bonding. Kumar et al.’s study [16] emphasized the significance of infill density and post-processing techniques on flexural strength. The results showed that annealed CFPETG at 100% infill density exhibited a notable increase in flexural strength compared to annealed PETG, with an improvement of approximately 18%. Ferreira et al. [17] characterized reinforced PETG, showing enhancements in flexural properties. Flexural tests demonstrated a 191.38% increase in flexural modulus and a 5.14% increase in strength, indicating improved interlayer adhesion. Srinidhi et al. [18] examined the influence of infill patterns (such as grid, honeycomb, rectilinear, and cubic) on PETG and CFPETG FFF-printed components. The results showed substantial enhancements in flexural strength, particularly in annealed grid pattern samples, with a 9% increase (73.5 MPa for PETG, 81 MPa for CFPETG). Panneerselvam et al. [19] analyzed the flexural strength of PETG, which was produced with FFF. The results revealed a notable increase in flexural strength with higher layer height, showcasing the significant improvement of the samples. SN ratio analysis emphasized layer height’s direct impact, yielding a 15% increase in flexural strength with optimized parameters. Fauntas et al. [20] conducted experiments for PETG by varying the FFF slicing parameters, resulting in the flexural strength changing between 12.5 MPa and 27.8 MPa. Optimal strength (27.8 MPa) was observed with a deposition angle parallel to the *x*-axis. High infill density (100%) yielded better strength (25.6 MPa), while lower densities coupled with lofty layer heights (80%, 0.3 mm) provided 20.3 MPa strength. Durgashyam et al. [21] explored how process parameters like layer height, feed rate, and infill density influence the mechanical properties of PETG material fabricated using the FFF technique. In their study, the infill density varied between 60–80%, and the highest flexural strength (77.65 MPa) was obtained at a 60% infill density. Taguchi analysis showed that higher flexural strength values can be obtained for 60% infill density compared to 70% and 80%. Based on the study of Durgashyam et al. [21], and to save weight, the infill density was kept constant at 50% in our current study. Guessasma et al. [22] analyzed the printability and tensile performance of 3D-printed PETG, demonstrating significant improvements in mechanical properties with optimized printing parameters. Another study by Hanon et al. [23] evaluated the anisotropy of different raster directions, spatial orientations, and fill percentages in 3D-printed PETG, highlighting the material’s versatility and potential for various applications. These recent advancements emphasize the growing interest and potential in optimizing PETG for diverse applications through material modifications and advanced printing techniques.

Three-dimensional printing technologies, especially the FFF method, are of great interest in optimizing the mechanical properties of polymers. Studies in this field show that careful optimization of printing parameters can significantly improve print quality and mechanical properties. Deswal et al. [24] optimized key process parameters to improve the dimensional accuracy of FFF printing devices. Hybrid statistical tools such as RSM-Genetic Algorithm (RSM-GA), Artificial Neural Network (ANN), and ANN-GA were used. Srinivasan et al. [25] applied RSM to predict the tensile strength of ABS parts printed with FFF, which significantly improved mechanical properties by determining the optimum printing parameters. Selvam et al. [26] used particle swarm optimization (PSO) to enhance the strength of AM components. They improved part performance with carbon fiber reinforcement and bio-inspired interlock sutures. Saad et al. [27] used RSM, particle search algorithms (PSO), and symbiotic organism search (SOS) algorithms to optimize the surface roughness in FDM 3D printing and achieved significant improvements in surface quality. Naveed and Anwar [28] applied experimental techniques and ANOVA-based statistical analysis to optimize 3D printing parameters to determine the optimal parameters for high-quality and consistent 3D-printed components. Das et al. [29] investigated the crystallization and rheological behavior of short carbon fiber-reinforced polyamide 6 (CF-PA6) filaments in the process of melt layer-by-layer deposition. The effects of printing parameters such as layer thickness, raster angle, and filler pattern on tensile properties were evaluated using the Taguchi method. Moradi et al. [30] investigated the process parameters of FFF-printed nylon parts. The effects of layer thickness, filler percentage, and number of contours were evaluated and optimized using RSM. Layer thickness was found to be the most influential parameter. Tunçel et al. [31] used the Taguchi method and GRA to optimize FFF parameters for 30% ceramic-reinforced composite PLA material. The results increased the tensile strength by 20.55% and reduced the production time by 43.75%. According to the literature, traditional optimization techniques such as the Taguchi method have effectively determined the appropriate printing parameters, but multi-objective optimization techniques such as RSM, GA, GRA, PSO, and ANN have been used for more complex materials and processes.

Recently, there has been a significant focus among researchers to investigate the mechanical properties of materials produced by FFF technology, a trend that continues to grow in importance. The literature review revealed a gap in research regarding the comprehensive analysis of the flexural performance of FFF components fabricated using PETG, which served as the driving force behind this study. This research aims to increase the durability and reliability of structural components used in automotive, aerospace, and medical industries by improving the flexural performance of PETG materials. The findings may enable the production of lighter and more durable parts in these fields. The current research proposes a parametric optimization study using RSM for the FFF fabrication of PETG material to improve the performance and applicability of 3D-printed objects in specific use cases. In particular, it focuses on determining the optimal FFF process parameters that result in the superior flexural performance of 3D-printed PETG parts. The study considers three key process parameters: layer height, print speed, and nozzle temperature. Flexural performance responses were evaluated using a three-point flexure test, including flexural strength, modulus, ultimate flexural toughness, flexural toughness at 5% strain, and strain.

## 2. Materials and Methods

**Figure 1:**
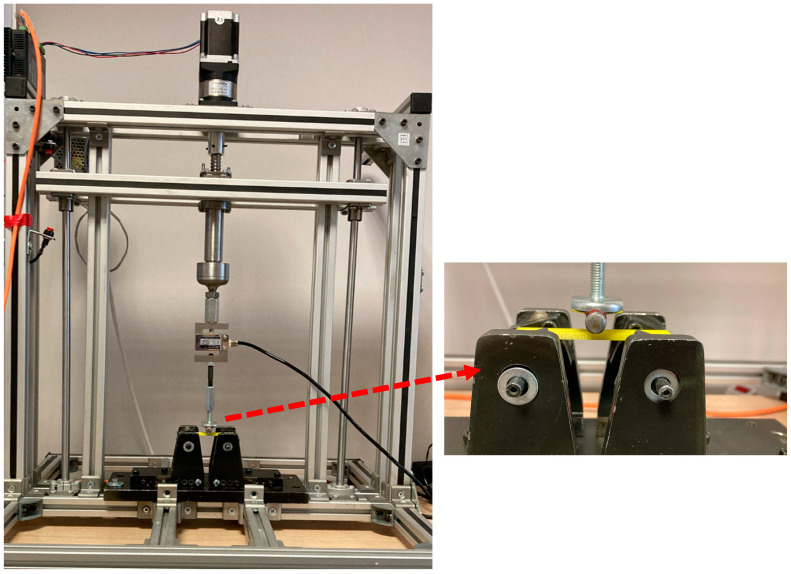
Test device for 3-point flexural tests.

The three-point flexural samples were produced using the Creality Ender 3-S1 Pro printer (Shenzen, China), which prints using the FFF method and has a nozzle diameter of 0.4 mm. The printing process used a Creality PETG filament with a diameter of 1.75 mm and a density of 1.27 g/cm^3^. The experimental design and the RSM analyses were conducted using Minitab, Version 20.3. The flexure tests were performed on a custom-built table-top testing machine designed for low-strength materials (Figure 1). The device is controlled by an Atmega2560 microcontroller (Microchip, Chandler, AZ, USA) and uses C++ for its software development. The user interface software was developed in C# (Microsoft Visual Studio 2022). Although the test device has a maximum load-carrying capacity of up to 1.5 kN, a 0.3 kN capacity load cell (Tedea, Shaanxi, China) is used to get more accurate measurements. A stepper motor was used to drive the loading head. This allowed the system to be controlled using open-loop control. The configuration settings for the motor drivers were set to 1600 pulses per revolution with a screw pitch and reduction ratio of 5, allowing precise loading head positioning in increments as small as 1/1600 mm. Displacement data acquisition involved tracking the position of the loading head. The data-acquisition card, which employed an ADS1256 from Texas Instruments, USA, was configured to operate at a sampling rate of 30 data points per second.

Three-point flexural test samples were prepared by ISO 178 standards using the Solidworks 2020 computer-aided design program [32,33]. Samples for this standard have dimensions of 80 mm × 10 mm × 4 mm. The Cura 5.5.0 software was used for slicing. The parameters that remained constant during the printing processes are provided in Table 1.

RSM is a method for statistically analyzing engineering problems to model the relationships among multiple variables and facilitate improvements in design and manufacturing processes [25,34]. The variables identified in this study affecting the responses to be used in RSM modeling were layer height, print speed, and nozzle temperature (Table 2). Three levels of layer height were considered: 0.15 mm, 0.20 mm, and 0.25 mm. The printing speed varied over a wide range, equal to 20 mm/s, 50 mm/s, and 80 mm/s. Additionally, three distinct nozzle temperatures were investigated: 230 °C, 240 °C, and 250 °C. Throughout the experiments, the parameters outlined in Table 1 remained constant.

**Figure 2:**
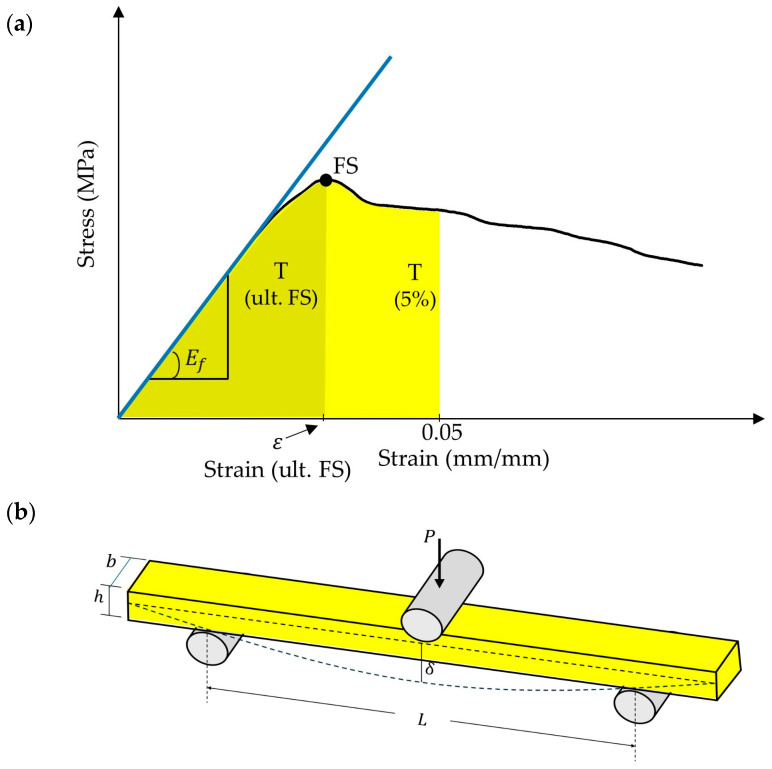
(**a**) A stress–strain graph of a flexural sample. (FS: flexural strength, $E_{f}$ flexural modulus, *T*: toughness) (**b**) schematic diagram of a sample under a three-point flexure.

Critical responses such as flexural strength, flexural modulus, flexural toughness at ultimate flexural strength, flexural toughness at 5% strain, and strain at ultimate flexural strength are depicted in Figure 2a below. Also, a schematic representation of the flexural experiment is provided in Figure 2b.

The relevant formulas used in the calculations are shown in the equations below (Equations (1)–(4)) [35,36]. Flexural strength (σ) and flexural strain (ε) can be obtained as follows:

(1) $$\sigma =\frac{3\mathrm{PL}}{2bh^{2}}$$

(2) $$\varepsilon =\frac{6\delta h}{L^{2}}$$

Here, P, δ, L, b, and h represent load, deflection of the center point of the beam, span length, width, and thickness of the test beam, respectively. The flexural modulus ($E_{f}$) can also be calculated by the following equation:

(3) $$E_{f}=\frac{L^{3}\theta }{4bh^{3}}$$

where θ is the slope obtained by considering two points in the elastic region of the load-deflection curve. Flexural toughness up to ultimate stress and 5% strain points have been calculated for assessment, as shown in the equation below.

(4) T=∫σdε

## 3. Results and Discussions

Flexural tests are essential to understand the material’s behavior under bending and its resistance to loads from different directions. Flexural properties are preferred in this study because 3D-printed parts are often subjected to complex loading conditions, and these tests better reflect the material’s performance under actual conditions of use. Furthermore, flexural tests are critical in understanding interlayer bonding problems, which are especially common in AM methods.

### 3.1. Experimental Results

**Figure 3:**
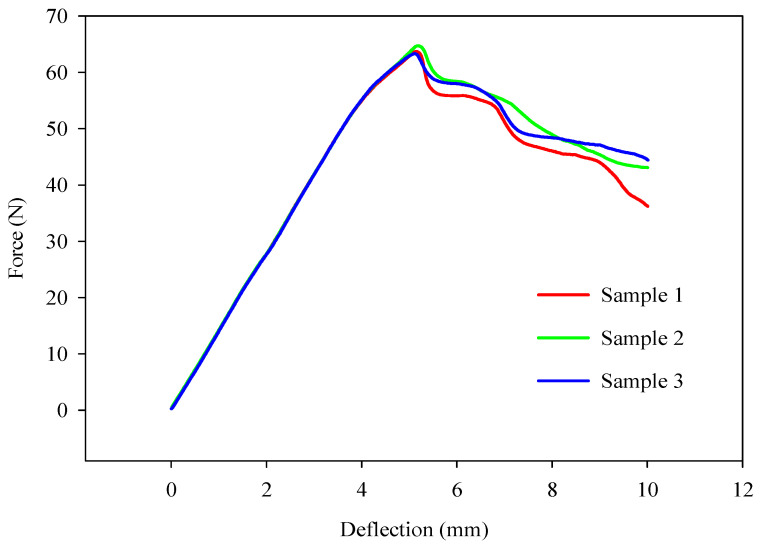
Representative bending force-deflection curves.

Three bending force-deflection curves with the same manufacturing process parameters are shown in Figure 3 as representative curves. This pattern was observed across other experimental groups as well. When repeated tests yield highly similar results, the standard deviation within the experimental groups will be low. This indicates reliable outcomes and suggests that fewer experiments may be necessary for a robust experimental design. So, Box–Behnken design type, which requires fewer experiments, was chosen for the response surface analysis [37].

Table 2 presents the input parameters, along with their corresponding levels. Additionally, Table 3 provides details regarding the responses and the Box–Behnken design employed. The RSM model was generated using the Minitab 20.3 software. Table 3 illustrates 15 experiments conducted, each representing different combinations of input parameters.

### 3.2. Anova Results

ANOVA provides quantitative data on the effects of the input parameters and their interactions with the responses [38]. Table 4, Table 5 and Table 6 show the ANOVA results for flexural strength, flexural modulus, flexural toughness for ultimate flexural strength, flexural toughness for 5% strain, and strain responses at ultimate flexural strength. The ANOVA results calculate two critical values. A 95% confidence level was chosen in the model setup [39]. The higher the F-values, the more influential the input parameter is on the response [40]. The most effective parameter on flexural modulus (79.38%), flexural toughness for ultimate flexural strength (67.36%), and strain at ultimate flexural strength (73.23%) is layer height. Nozzle temperature is the most influential parameter on flexural strength (56.64%) and flexural toughness for 5% strain (33.62%). On the other hand, print speed is generally less influential on responses.

RSM significantly decreases the number of experiments and can detect factor interactions based on the selected model, necessitating the examination of experimental results using a second-order polynomial response-regression model for precise prediction. The second-order equations of the output responses (flexural strength, flexural modulus, flexural toughness for ultimate flexural strength, flexural toughness for 5% strain, and strain at ultimate flexural strength according to the input parameters) are provided in Table 7. An explanation of the system and the necessary equations can be found in the study by Yaman et al. [41].

The list of R^2^ values (coefficient of determination of the equation) that is used in evaluating the models is provided in Table 8. All output parameters have R^2^ values greater than 0.99, which is indicative of a good fit. Similarly, the adjusted R^2^ and predicted R^2^ values, which indicate the accuracy of the model, were also found to be acceptably high. According to the results obtained from the model, R^2^ values were determined as 99.33%, 99.40%, 99.32%, 99.46%, and 99.12% for flexural strength, flexural modulus, and flexural toughness for ultimate flexural strength, flexural toughness for 5% strain, and strain at ultimate flexural strength, respectively, as shown in Table 8. The detailed formulas used to explain R^2^, adjusted R^2^, and predicted R^2^ are presented in the Çalhan et al. study [42].

### 3.3. RSM Results

**Figure 4:**
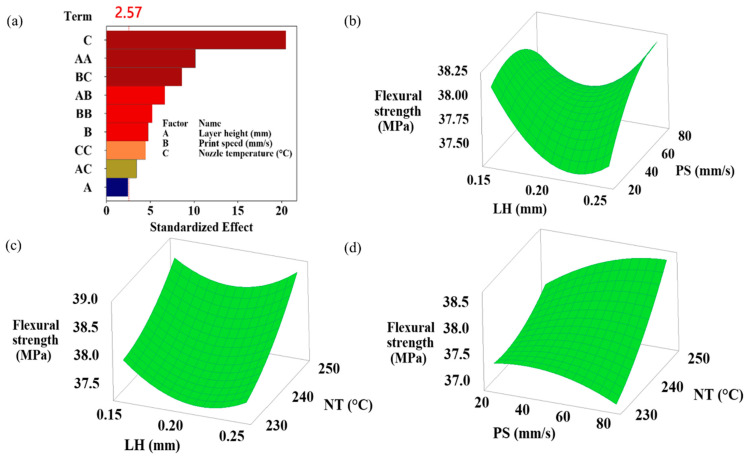
(**a**) Pareto chart and (**b**–**d**) simultaneous impacts of layer height, print speed, and nozzle temperature on flexural strength.

**Figure 5:**
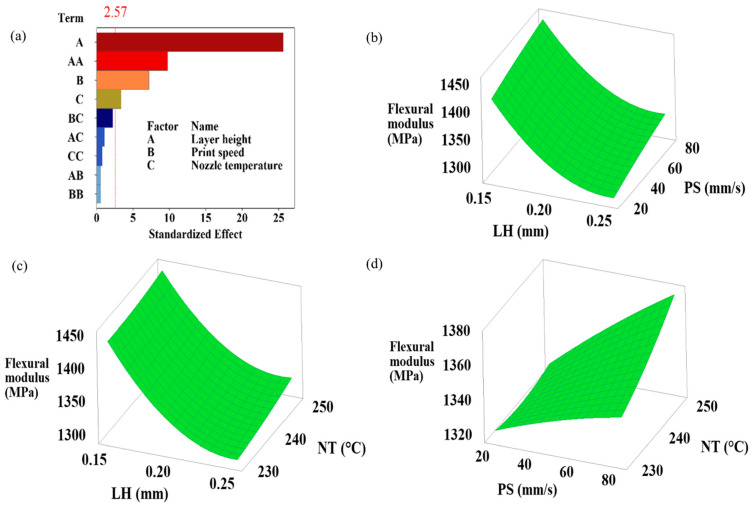
(**a**) Pareto chart and (**b**–**d**) simultaneous impacts of layer height, print speed, and nozzle temperature on flexural modulus.

**Figure 6:**
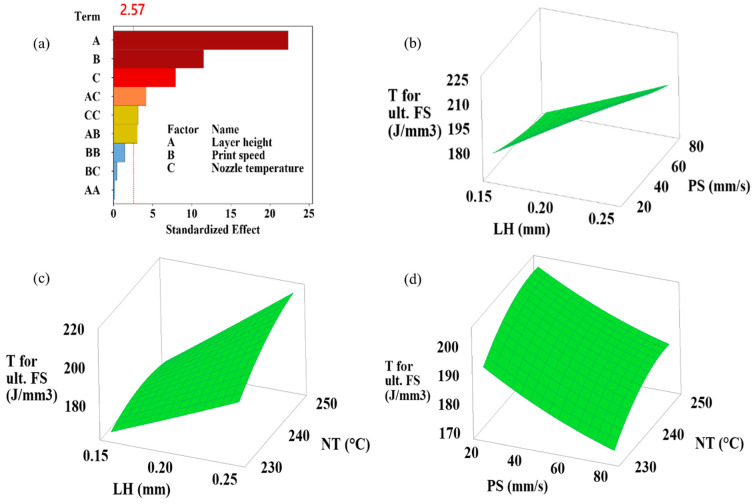
(**a**) Pareto chart and (**b**–**d**) simultaneous impacts of layer height, print speed, and nozzle temperature on flexural toughness for ultimate flexural strength.

**Figure 7:**
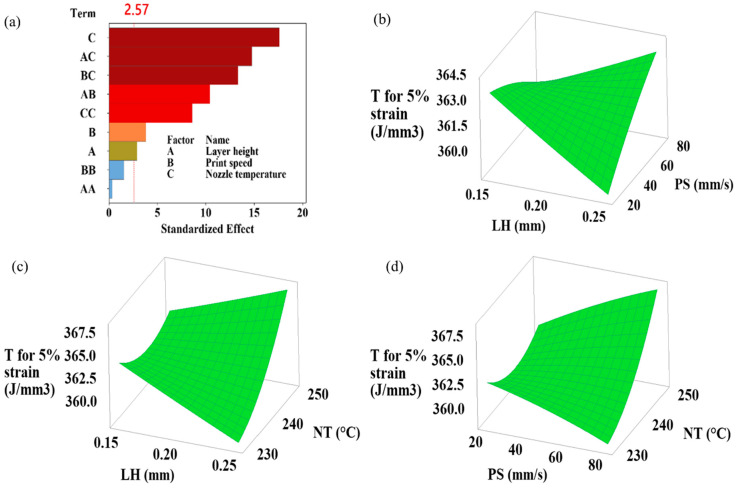
(**a**) Pareto chart and (**b**–**d**) simultaneous impacts of layer height, print speed, and nozzle temperature on flexural toughness for 5% strain.

**Figure 8:**
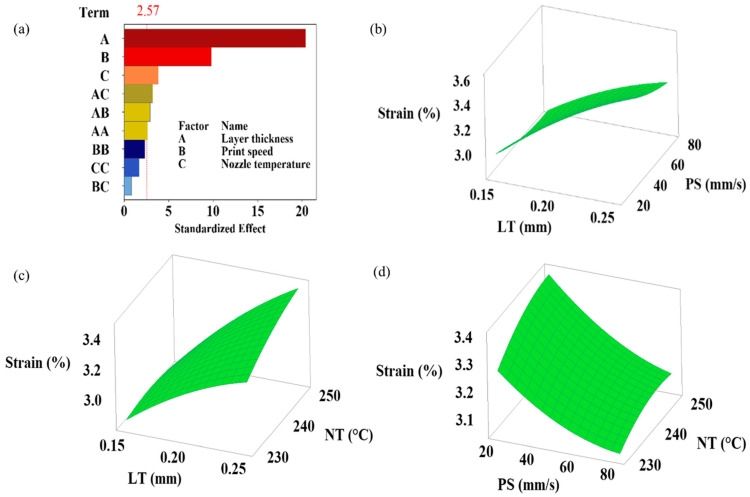
(**a**) Pareto chart and (**b**–**d**) simultaneous impacts of layer height, print speed, and nozzle temperature on strain at ultimate flexural strength.

This study highlights the critical presence of interaction effects, showing how the combined effects of different input parameters exceed (or fall short of) their individual effects and simultaneously influence at least one output response. Understanding how the processing parameters of a 3D printer affect the flexural performance of printed samples depends on recognizing these interactions [27]. The simultaneous effects of layer height, print speed, and nozzle temperature variations on flexural strength, flexural modulus, flexural toughness at ultimate flexural strength, flexural toughness at 5% strain, and strain at ultimate flexural strength are shown in the 3D plots generated by RSM in Figure 4, Figure 5, Figure 6, Figure 7 and Figure 8. Pareto plots are also shown in Figure 4, Figure 5, Figure 6, Figure 7 and Figure 8 to better evaluate the individual and combined effects of each selected parameter.

#### 3.3.1. Flexural Strength Results

Flexural strength provides information on the strength of the material using the ultimate load generated during the flexure test. Figure 4 shows the Pareto chart and the effects of layer height, print speed, and nozzle temperature on flexural strength. Figure 4a presents the Pareto plot of flexural strength. According to the Pareto plot of flexural strength, the order of influence is nozzle temperature, layer height*layer height, and print speed*nozzle temperature. The ANOVA results in Table 4 reveal that this effect is caused by 56.64% nozzle temperature, 14.24% layer height*layer height, and 9.99% print speed*nozzle temperature. The *p*-value of layer height for flexural strength (0.056) is higher than 0.05, indicating that the effect of the layer height on flexural strength is insignificant. Figure 4b–d shows the simultaneous impacts of layer height, print speed, and nozzle temperature on flexural strength. Figure 4c,d illustrates flexural strength increases with increasing temperature. It was also observed that the compression speed of about 50 mm/s provided the highest flexural strength (Figure 4b,d). The highest flexural strength value was obtained as 38.91 MPa in Sample 3 with a 250 °C nozzle temperature, 50 mm/s print speed, and 0.25 mm layer height parameters (Table 3).

#### 3.3.2. Flexural Modulus Results

Flexural modulus is the stress ratio to the corresponding strain within elastic limits. Figure 5 shows the Pareto chart and 3D plots of flexural strength for different layer heights, print speeds, and nozzle temperature levels. Figure 5a shows that the maximum effect on the flexural modulus is due to the layer height. According to Table 4, where ANOVA results are displayed, the layer height has an effective rate of 79.38%, the layer height*layer height is 11.59%, and the print speed is 6.20%. Figure 5b–d shows the simultaneous effect of the variables on the flexural modulus. Flexural modulus increased with the increase in print speed and nozzle temperature. However, it decreased with an increase in layer height. The highest flexural modulus value was 1468.18 MPa for Sample 8, with the parameters of 0.15 mm layer height, 80 mm/s printing speed, and 240 °C nozzle temperature (Table 3).

#### 3.3.3. Flexural Toughness for Ultimate Flexural Strength Results

Analyzing the Pareto chart in Figure 6a, the order of the effect of the parameters on flexural toughness for ultimate flexural strength is layer height, print speed, and nozzle temperature. The effect of the interactions of the parameters remained small. In the ANOVA table in Table 5, it was previously shown that the linear effect of the parameters was very high at 93.96%. In the same table, it was also seen that the layer height reached 67.36%. Analyzing the interactions in Figure 6b,c, it can be seen that increasing the layer height causes an increase in toughness. While the rise in nozzle temperature increased the toughness, as did the increase in layer height, while the toughness decreased with the increase in print speed (Figure 6b,c). The highest toughness was obtained as 227.11 J/mm^3^ in the sample coded 6. This sample has 0.25 mm layer height, 20 mm/s print speed, and 240 °C nozzle temperature parameters (Table 3). The lowest toughness was obtained as 166.26 J/mm^3^ in Sample 7. This sample has 0.15 mm layer height, 50 mm/s print speed, and 230 °C nozzle temperature parameters (Table 3). Sample 6 has a 36.6% higher toughness value than Sample 7. When the toughness values in Table 3 are examined, the results for flexural toughness reach a 5% strain change over a much narrower range than the flexural toughness at ultimate strain and converge.

#### 3.3.4. Flexural Toughness for 5% Strain Results

Figure 7 shows the Pareto chart and 3D plots of flexural toughness for 5% strain according to layer height, print speed, and nozzle temperature. As shown in Figure 7a, the maximum effect on flexural toughness at 5% strain is due to nozzle temperature and the interaction of nozzle temperature with layer height and print speed. According to Table 5, which shows the results of ANOVA, the nozzle temperature has an effective rate of 33.62%, the layer height is 0.91%, and the printing speed is 1.57%. The nozzle temperature/layer height interaction had an effect of 23.68%, while the print-speed interaction had an impact of 19.28%. Flexural toughness at 5% strain increased with increasing nozzle temperature. In particular, for the 0.25 mm layer height and 80 mm/s print speed parameters, the increase in flexural toughness at 5% strain was more significant with increasing nozzle temperature (Figure 7c,d). Changes in layer height and print-speed parameters have mixed effects regarding the increases and decreases in flexural toughness at 5% strain (Figure 7b–d). The highest flexural toughness for the 5% strain value was obtained as 368.48 J/mm^3^ at 0.20 mm layer height, 80 mm/s print speed, and 250 °C nozzle temperature.

#### 3.3.5. Strain at Ultimate Flexural Strength Results

Figure 8 shows the simultaneous effects of layer height, print speed, and nozzle temperature on strain at ultimate flexural strength. In addition, the degree of influence of the variables on the strain (%) is provided in Figure 8a. The Pareto plot for strain shows that layer height is the most influential parameter, followed by print speed and nozzle temperature. The ANOVA results show that the contribution of layer height to strain is 73.23%, the print speed is 16.91%, and the nozzle temperature is 2.62%. The simultaneous effects of the variables show that increasing layer height promotes an increase in strain while increasing print speed promotes a decrease (Figure 8b–d). Nozzle temperature has a limited effect on strain. Based on the test results in Table 3, the highest strain at the ultimate flexural strength value is 3.66% for the parameters of 0.25 mm layer height, 20 mm/s print speed, and 240 °C nozzle temperature.

### 3.4. RSM Prediction vs. Experimental Results

This section draws a contrast to assess the degree of agreement between the predictions generated by the regression equations and the 15 experiments used to construct the RSM model. Table 9, Table 10 and Table 11 compare the prediction results of the RSM model and the experimental results, along with the corresponding error percentages for each output response. When the error means between the test results and the RSM predictions are analyzed, the results are in good agreement with very low error percentages. The best fits were found for flexural toughness at 5% strain with an error of 0.13%, while the worst fit was found for flexural modulus with an error of 0.83%.

### 3.5. Optimization and Validation of Responses

**Figure 9:**
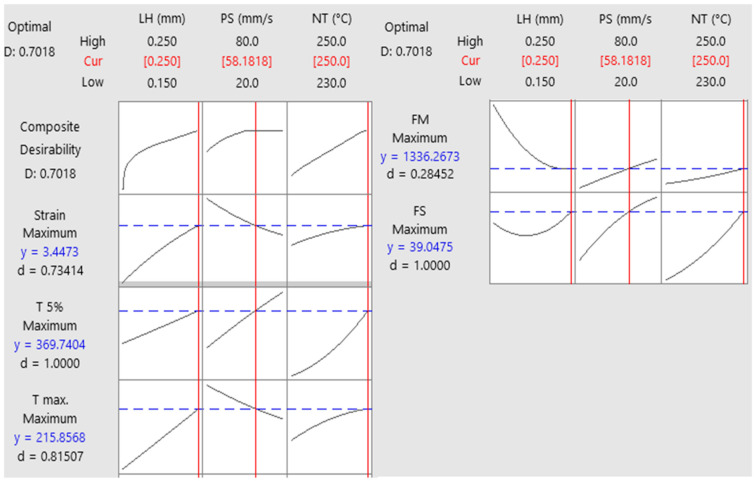
Optimization results. (FS: flexural strength, FM flexural modulus, T: toughness).

In this study, the multiple responses listed in Table 11 were optimized using the response optimizer according to the stated objectives and as part of the RSM analyses. The main objective was to find the maximum values for all responses. Figure 9 shows the results of the RSM optimizer. The optimum values for strain, toughness at 5% strain, toughness at ultimate FS, flexural modulus, and flexural strength were obtained as 3.45%, 369.74 J/mm^3^, 215.86 J/mm^3^, 1336.27 MPa, and 39.05 MPa for optimum input parameters of 0.25 mm layer height, 58.18 mm/s print speed, and 250 °C nozzle temperature, respectively. In optimization studies, the desirability value is expected to be close to 1. The value of 0.7018 in the present study shows an acceptable desirability value. Nevertheless, verification experiments were conducted to prove the accuracy of the optimization study, and the results obtained were compared with those obtained as a result of the optimization. Table 12 shows the optimization results, the validation experiment results, and the error rates between them. The results of the validation experiments were above the predicted values for all outputs. In the verification experiments, for the optimal parameters 3.50%, 381.47 J/mm^3^, 218.22 J/mm^3^, 1344.60 MPa, and 39.55 MPa strain, toughness for 5% strain, toughness for ultimate FS, flexural modulus, and flexural strength, respectively, were obtained. When the error rates are analyzed, it can be seen that all of them are less than 1.5% except for toughness at 5% strain. The lowest error rate was obtained in flexural modulus with 0.62%, while the highest error occurred in toughness at 5% strain with 3.17%.

## 4. Conclusions

The study focused on optimizing FFF process parameters for PETG material and evaluated various mechanical responses such as flexural strength, flexural modulus, and flexural toughness through three-point flexure tests. Key process parameters included layer height, print speed, and nozzle temperature. This study used Box–Behnken design within RSM to create models representing the relationships between variables and responses. The main results obtained are presented below.

According to the results, increasing the layer height and nozzle temperature increased the mechanical properties of PETG material under the flexural load. Increasing layer height decreased printing time and thermal stress, while increasing nozzle temperature led to better fusion of the layers, resulting in more robust and high-integrity final products. The ANOVA analysis revealed the quantitative effects of input parameters and their interactions with responses. Layer height predominantly affected flexural modulus (79.38%), flexural toughness for ultimate flexural strength (67.36%), and strain at ultimate flexural strength (73.23%). Conversely, nozzle temperature significantly impacted flexural strength (56.64%) and flexural toughness for 5% strain (33.62%).

Experimental data were used to construct second-order polynomial response-regression models that yielded high coefficients of determination (R^2^) ranging from 99.12% to 99.46%.

Utilizing RSM, the optimal parameters were determined: layer height of 0.25 mm, print speed of 58.18 mm/s, and nozzle temperature of 250 °C. Significant improvements in mechanical properties were observed: strain (3.50%), toughness at 5% strain (381.47 J/mm^3^), toughness at ultimate flexural strength (218.22 J/mm^3^), flexural modulus (1344.60 MPa), and flexural strength (39.55 MPa).

Desirability was high at 0.7018, validated by verification experiments closely matching predicted values with minimal errors (0.13% to 3.17%).

Future research could further explore additional 3D-printing parameters, such as different fill patterns, structure orientations, and post-processing techniques, to enhance the mechanical properties of FFF-printed PETG parts. Furthermore, evaluating the optimized parts’ long-term durability and environmental impact will provide valuable insights. Comparing the performance of FFF-printed PETG parts with commercial and conventionally produced PETG parts will also be helpful for broader practical applications and sustainability considerations in additive manufacturing.

## Figures and Tables

**Table 1:** Constant printing parameters of the flexural test samples.

	Units	Values
Nozzle diameter	mm	0.4
Table temperature	°C	70
Infill density	%	50
Infill pattern	-	Lines
Infill line directions	°	0/90
Build orientation	-	Flat
Wall thickness	mm	0.4
Top/Bottom thickness	mm	0.2
Fan speed	%	100

**Table 2:** Three-dimensional-printing process parameters and their levels.

No	Process Parameters	Units	Levels		
			−1	0	1
1	Layer height	mm	0.15	0.20	0.25
2	Print speed	mm/s	20	50	80
3	Nozzle temperature	°C	230	240	250

**Table 3:** The Box–Behnken design of experiments and the response of the outputs.

No	Layer Height (mm)	Print Speed (mm/s)	Nozzle Temperature (°C)	Flexural Strength (MPa)	Flexural Modulus (MPa)	Flexural Toughness for Ultimate Flexural Strength (J/mm^3^)	Flexural Toughness for 5% Strain (J/mm^3^)	Strain at Ultimate Flexural Strength (%)
1	0.15	20	240	38.10	1426.33	181.30	363.16	3.02
2	0.25	50	230	37.60	1305.33	193.64	358.04	3.25
3	0.25	50	250	38.91	1330.79	220.74	368.23	3.51
4	0.20	20	230	37.41	1327.91	195.21	363.22	3.30
5	0.25	80	240	38.23	1333.49	199.85	364.20	3.29
6	0.25	20	240	37.43	1283.81	227.11	358.87	3.66
7	0.15	50	230	37.92	1442.79	166.26	364.71	2.87
8	0.15	80	240	37.93	1468.18	168.63	360.69	2.86
9	0.15	50	250	38.73	1452.24	173.31	363.85	2.90
10	0.20	50	240	37.81	1341.34	191.58	362.08	3.20
11	0.20	80	230	36.96	1340.89	174.85	358.82	3.10
12	0.20	80	250	38.63	1374.79	185.92	368.45	3.12
13	0.20	50	240	37.73	1349.71	193.67	362.05	3.21
14	0.20	20	250	37.83	1329.43	204.17	362.88	3.38
15	0.20	50	240	37.67	1336.13	191.36	361.77	3.23

**Table 4:** ANOVA results of flexural strength and flexural modulus.

	Flexural Strength			Flexural Modulus		
	F-Value	*p*-Value	Contribution	F-Value	*p*-Value	Contribution
Model	81.78	0.000	99.33%	91.41	0.000	99.40%
Linear	149.55	0.000	60.54%	239.88	0.000	86.95%
LH-Layer height (mm)	6.16	0.056	0.83%	657.00	0.000	79.38%
PS-Print speed (mm/s)	22.74	0.005	3.07%	51.34	0.001	6.20%
NT-Nozzle temperature (°C)	419.74	0.000	56.64%	11.31	0.020	1.37%
Square	52.32	0.000	21.18%	32.27	0.001	11.69%
LH*LH	103.24	0.000	14.24%	95.18	0.000	11.59%
PS*PS	27.52	0.003	4.24%	0.27	0.628	0.04%
NT*NT	20.02	0.007	2.70%	0.55	0.493	0.07%
Two-way interaction	43.47	0.001	17.60%	2.08	0.221	0.075%
LH*PS	44.56	0.001	6.01%	0.28	0.619	0.03%
LH*NT	11.84	0.018	1.60%	1.17	0.329	0.14%
PS*NT	74.01	0.000	9.99%	4.79	0.080	0.58%
Error			0.67%			0.60%
Total			100%			100%

**Table 5:** ANOVA results of flexural toughness for ultimate flexural strength and flexural toughness for 5% strain.

	Flexural Toughness for Ultimate Flexural Strength			Flexural Toughness for 5% Strain		
	F-Value	*p*-Value	Contribution	F-Value	*p*-Value	Contribution
Model	81.38	0.000	99.32%	101.65	0.000	99.46%
Linear	230.98	0.000	93.96%	110.72	0.000	36.11%
LH-Layer height (mm)	496.77	0.000	67.36%	8.41	0.034	0.91%
PS-Print speed (mm/s)	132.91	0.000	18.02%	14.49	0.013	1.57%
NT-Nozzle temperature (°C)	63.25	0.001	8.58%	309.25	0.000	33.62%
Square	4.27	0.076	1.74%	26.33	0.002	8.59%
LH*LH	0.04	0.859	0.01%	0.11	0.755	0.00%
PS*PS	2.03	0.214	0.38%	2.38	0.183	0.53%
NT*NT	9.93	0.025	1.35%	74.07	0.000	8.05%
Two-way interaction	8.90	0.019	3.62%	167.90	0.000	54.76%
LH*PS	9.17	0.029	1.24%	108.54	0.000	11.80%
LH*NT	17.32	0.009	2.35%	217.82	0.000	23.68%
PS*NT	0.19	0.680	0.03%	177.33	0.000	19.28%
Error			0.68%			0.54%
Total			100%			100%

**Table 6:** ANOVA results of strain at ultimate flexural strength.

	Strain at Ultimate Flexural Strength		
	F-Value	*p*-Value	Contribution
Model	62.40	0.000	99.12%
Linear	175.22	0.000	92.77%
LH-Layer height (mm)	414.95	0.000	73.23%
PS-Print speed (mm/s)	95.84	0.000	16.91%
NT-Nozzle temperature (°C)	14.87	0.012	2.62%
Square	5.43	0.050	2.87%
LH*LH	6.98	0.046	1.29%
PS*PS	5.42	0.067	1.07%
NT*NT	2.90	0.150	0.51%
Two-way interaction	6.56	0.035	3.47%
LH*PS	8.62	0.032	1.52%
LH*NT	10.35	0.024	1.83%
PS*NT	0.70	0.440	0.12%
Error			0.88%
Total			100%

**Table 7:** Regression equations of responses. (LH: layer height, PS: print speed, and NT: nozzle temperature).

Regression Equations	
Flexural strength	154.3–130.8 LH–0.2562 PS–0.861 NT + 153.7 LH*LH–0.000220 PS*PS + 0.001692 NT*NT + 0.1617 LH*PS + 0.2500 LH*NT + 0.001042 PS*NT
Flexural modulus	4325–9333 LH–5.89 PS–15.7 NT + 15,018 LH*LH −0.00221 PS*PS + 0.0285 NT*NT +1.30 LH*PS + 8.00 LH*NT + 0.0270 PS*NT
Flexural toughness for ultimate flexural strength	−1819–1942 LH–0.461 PS + 17.55 NT + 94 LH*LH + 0.00198 PS*PS–0.0395 NT*NT–2.432 LH*PS + 10.02 LH*NT + 0.00176 PS*NT
Flexural toughness for 5% strain	1651- 1408.9 LH–2.204 PS–9.336 NT + 25.7 LH*LH–0.000334 PS*PS + 0.01677 NT*NT + 1.300 LH*PS + 5.525 LH*NT + 0.008308 PS*NT
Strain at ultimate flexural strength	−13.1–12.83 LH + 0.0101 PS + 0.1364 NT − 19.67 LH*LH + 0.000048 PS*PS −0.000317 NT*NT- 0.0350 LH*PS + 0.1150 LH*NT- 0.000050 PS*NT

**Table 8:** R^2^ values of responses from ANOVA results.

	R^2^ (%)	Adjusted R^2^ (%)	Predicted R^2^ (%)
Flexural strength	99.33	98.11	92.67
Flexural modulus	99.40	98.31	93.19
Flexural toughness for ultimate flexural strength	99.32	98.10	90.20
Flexural toughness for 5% strain	99.46	98.48	91.93
Strain at ultimate flexural strength	99.12	97.53	86.77

**Table 9:** Comparison of test and RSM responses for ultimate flexural strength, flexural modulus, and flexural toughness for ultimate FS.

Test No	Ultimate Flexural Strength (MPa)			Flexural Modulus (MPa)			Flexural Toughness for Ultimate FS (J/mm^3^)		
	Test	RSM	Error (%)	Test	RSM	Error (%)	Test	RSM	Error (%)
1	38.10	38.23	0.35	1426.33	1439.37	0.91	181.30	182.06	0.42
2	37.60	37.70	0.27	1305.33	1313.75	0.65	193.64	196.22	1.33
3	38.91	39.02	0.28	1330.79	1340.35	0.72	220.74	219.88	0.39
4	37.41	37.49	0.22	1327.91	1334.49	0.50	195.21	194.17	0.53
5	38.23	38.36	0.34	1333.49	1343.03	0.72	199.85	200.32	0.23
6	37.43	37.62	0.52	1283.81	1301.39	1.37	227.11	227.24	0.06
7	37.92	38.08	0.41	1442.79	1455.83	0.90	166.26	168.36	1.26
8	37.93	38.00	0.19	1468.18	1473.21	0.34	168.63	169.73	0.66
9	38.73	38.89	0.42	1452.24	1466.43	0.98	173.31	171.97	0.77
10	37.81	37.87	0.15	1341.34	1353.70	0.92	191.58	192.82	0.65
11	36.96	37.12	0.44	1340.89	1356.03	1.13	174.85	173.49	0.78
12	38.63	38.81	0.47	1374.79	1390.83	1.17	185.92	188.19	1.22
13	37.73	37.87	0.37	1349.71	1353.70	0.30	193.67	192.82	0.44
14	37.83	37.93	0.27	1329.43	1336.89	0.56	204.17	206.75	1.27
15	37.67	37.87	0.53	1336.13	1353.70	1.31	191.36	192.82	0.76
Mean error (%)			0.35			0.83			0.72

**Table 10:** Comparison of test and RSM responses for flexural toughness for 5% strain and strain for ultimate FS.

Test No	Flexural Toughness for 5% Strain (J/mm^3^)			Strain for Ultimate FS (%)		
	Test	RSM	Error (%)	Test	RSM	Error (%)
1	363.16	364.02	0.24	3.02	3.03	0.20
2	358.04	358.68	0.18	3.25	3.29	1.26
3	368.23	368.88	0.18	3.51	3.50	0.27
4	363.22	363.45	0.06	3.30	3.29	0.27
5	364.20	364.25	0.01	3.29	3.30	0.16
6	358.87	359.36	0.14	3.66	3.65	0.38
7	364.71	364.97	0.07	2.87	2.89	0.72
8	360.69	361.11	0.12	2.86	2.88	0.87
9	363.85	364.12	0.07	2.90	2.87	1.02
10	362.08	362.42	0.09	3.20	3.22	0.59
11	358.82	359.46	0.18	3.10	3.08	0.80
12	368.45	369.13	0.18	3.12	3.14	0.64
13	362.05	362.42	0.10	3.21	3.22	0.28
14	362.88	363.15	0.07	3.38	3.42	1.06
15	361.77	362.42	0.18	3.23	3.22	0.34
Mean error (%)			0.13			0.59

**Table 11:** RSM optimization details.

Response	Goal	Lower	Target	Upper	Weight	Importance
Strain	Maximize	2.86	3.66	3.66	1	1
Toughness for %5 strain	Maximize	358.04	368.45	368.45	1	1
Toughness for ultimate FS	Maximize	166.26	227.11	227.11	1	1
Flexural modulus	Maximize	1283.81	1468.18	1468.18	1	1
Flexural strength	Maximize	36.96	38.91	38.91	1	1

**Table 12:** Validation test results. (FS: flexural strength, FM flexural modulus, T: toughness).

Layer Height (mm)	Print Speed (mm/s)	Nozzle Temperature (°C)		FS (MPa)	FM (MPa)	T Ultimate (J/mm^3^)	T 5% (J/mm^3^)	Strain (%)	Desirability
0.25	58.18	250	Optimum	39.05	1336.27	215.86	369.74	3.45	0.7018
			Actual	39.55	1344.60	218.22	381.47	3.50	
			%Error	1.28	0.62	1.09	3.17	1.45	

## Data Availability

Data are contained within the article.
